# Non-communicable diseases, digital education and considerations for the Indian context – a scoping review

**DOI:** 10.1186/s12889-024-18765-7

**Published:** 2024-05-10

**Authors:** Anup Karan, Suhaib Hussain, Lasse X Jensen, Alexandra Buhl, Margaret Bearman, Sanjay Zodpey

**Affiliations:** 1 Public Health Foundation of India, New Delhi, India; 2 Department of Public Health, University of Copenhagen, Copenhagen, Denmark; 3 Centre for Research on Assessment and Digital Learning, Deakin University, Melbourne, Australia

**Keywords:** Health professions education, Non-communicable diseases (NCD), India, Digital education, Scoping review

## Abstract

**Introduction:**

The increasing ageing of the population with growth in NCD burden in India has put unprecedented pressure on India’s health care systems. Shortage of skilled human resources in health, particularly of specialists equipped to treat NCDs, is one of the major challenges faced in India. Keeping in view the shortage of healthcare professionals and the guidelines in NEP 2020, there is an urgent need for more health professionals who have received training in the diagnosis, prevention, and treatment of NCDs. This paper conducts a scoping review and aims to collate the existing evidence on the use of digital education of health professionals within NCD topics.

**Methods:**

We searched four databases (Web of Science, PubMed, EBSCO Education Research Complete, and PsycINFO) using a three-element search string with terms related to digital education, health professions, and terms related to NCD. The inclusion criteria covered the studies to be empirical and NCD-related with the target population as health professionals rather than patients. Data was extracted from 28 included studies that reported on empirical research into digital education related to non-communicable diseases in health professionals in India. Data were analysed thematically.

**Results:**

The target groups were mostly in-service health professionals, but a considerable number of studies also included pre-service students of medicine (*n* = 6) and nursing (*n* = 6). The majority of the studies included imparted online learning as self-study, while some imparted blended learning and online learning with the instructor. While a majority of the studies included were experimental or observational, randomized control trials and evaluations were also part of our study.

**Discussion:**

Digital HPE related to NCDs has proven to be beneficial for learners, and simultaneously, offers an effective way to bypass geographical barriers. Despite these positive attributes, digital HPE faces many challenges for its successful implementation in the Indian context. Owing to the multi-lingual and diverse health professional ecosystem in India, there is a need for strong evidence and guidelines based on prior research in the Indian context.

**Supplementary Information:**

The online version contains supplementary material available at 10.1186/s12889-024-18765-7.

## Introduction

Non-communicable diseases (NCDs) kill 41 million people each year. Of these deaths, more than 15 million happen to people between the ages of 30 and 69 years, and the vast majority of these “premature” deaths occur in low- and middle-income countries (LMICs) [1]. It is estimated that by 2030 the share of NCDs in global total mortality will be 69% – a dramatic rise from 59% in 2002 [2]. Although the burden of NCDs continues to increase across all regions of the world, it disproportionately affects poorer regions [3], with almost 80% of NCD-related deaths occurring in LMICs [4].

This shift is largely driven by demographical and epidemiological transitions, coupled with rapid urbanization and nutritional transitions in LMICs [5].

With approximately six million annual deaths from NCDs, India presents an important case study with respect to these challenges [6]. Similar to many other LMICs, India is experiencing a rapid health transition with a rising burden of NCDs now surpassing the burden of communicable diseases [7]. In India, NCDs such as cardiovascular diseases, cancer, chronic respiratory diseases, and diabetes are estimated to account for around 63% of all deaths, thus making them the leading causes of death [6]. This NCD burden has severe implications for the healthcare system. In particular, the shortage of skilled health professionals, i.e. medical specialists, nurses, and other professionals equipped to treat NCDs, presents a serious challenge [8]. The inadequacy of educational institutions to impart quality medical and nursing education has been one of the main reasons for the health workforce shortage [8]. In a recent study, the number of Indian doctors and nurses/midwives was estimated at 0.80 million and 1.40 million, with a density of 6.1 and 10.6, respectively, per 10,000 population. The numbers further drop to 5.0 and 6.0 per 10,000 population, respectively, after accounting for the adequate qualifications [9, 10]. All these estimates are well below the WHO threshold of 44.5 doctors, nurses and midwives per 10,000 population [11]. The study also highlights the highly skewed distribution of the health workforce across states, rural–urban and public–private sectors. The skewed distribution of the health workforce across India means that this shortage is even more grave in rural and remote areas [9, 10]. The revised guidelines of the National Programme for Prevention and Control of Non-Communicable Diseases (NP-NCD), are a welcome strategy in the prevention and control of NCDs [12]. The focus of the guidelines on health promotion, early diagnosis and screening, and capacity building of healthcare professionals will definitely push for increased attention to the management of NCDs and how this relates to the pre- and in-service training needs of health professionals. In addition, the recent establishment of Health and Wellness Centres (HWC) in managing NCDs and achieving UHC is an excellent response to the changing demographic and epidemiological profile in India. However, this initiative is not without challenges, with a major challenge being the need to build human resource capacity with a continued need for training [13, 14]. Although some states have conducted specific training programs to improve the capacity and address the issue, the lack of training modules for NCD management remains an important challenge to be addressed [14]. The need to strengthen the HWCs through adequate financing, human resources, and logistics for medicines and technology, especially in hard geographical areas, is an area to be focussed upon [13].

The National Education Policy (NEP) 2020 by the government of India has highlighted the role of digital education in training and continuing education [15]. Digital education is defined as an act of teaching and learning by means of digital technologies involving a multitude of educational approaches, concepts, methods, and technologies [16]. The NEP 2020 focuses attention on implementing and strengthening multidisciplinary, inclusive and technology-based learning that is accessible to all. With a large geographical and cultural diversity in India, meeting this need has proven to be a challenge to India’s existing systems of health professions education (HPE). Hence, the use of technology in education is proposed as a way to access remote areas and bypass geographical barriers [15].

Although the NEP 2020 has some aspirational objectives, there is a lack of specific knowledge regarding the digital education of health professionals in India. A recent review of Indian research in digital health professions education found that the body of literature is very limited and that the studies that do exist tend to take the form of evaluations of local educational interventions rather than more systematic contributions to research-based knowledge [17].

Considering the scarcity of empirical evidence related to digital education and training of health professionals regarding NCDs, it is relevant to look outside of India and explore what research may have been done in other contexts.

### Objective

Digitalization of education may help us address the urgent need for more health professionals who have received training in the diagnosis, prevention, and treatment of NCDs. However, it is still unclear what constitutes best practice in NCD-related digital education, and how experiences from across the world are relevant to the Indian context.

The objective of the present paper is to conduct a scoping review of the published research examining the digital education of health professionals within NCD topics. More specifically the paper aims to: (i) assess the strengths and weaknesses of the digital teaching-learning practices described in the literature; and (ii) discuss the findings in relation to the Indian context.

## Methods

The scoping review methodology is appropriate for exploring the extent of research activity within a topic where the literature is limited and disorganized. With a more flexible approach than what is known from systematic reviews, the scoping methodology can provide an overview of what kinds of evidence exist and help inform future research [18].

To identify relevant publications, we searched four research databases (Web of Science, PubMed, EBSCO Education Research Complete, and PsycInfo). This was done with a search string consisting of three elements, namely terms related to digital education (*n* = 174), terms related to health professions (*n* = 30), and terms related to NCD (*n* = 36). The search string with all terms is included in the online supplementary material.

The search produced 1032 hits combined from all the databases (Web of Science: 443; PubMed: 259; EBSCO Education Research Complete: 118; PsycInfo: 212). When searching, we did not limit the search to any specific time frame, but subsequently, we opted to exclude papers published before 2017. This was decided to ensure that the included papers reported on interventions that represent current digital technologies. After removing duplicates and papers published before 2017, we had 463 documents. These documents were imported into the online review tool Covidence, which was used to manage the screening and data extraction processes.

**Fig. 1:**
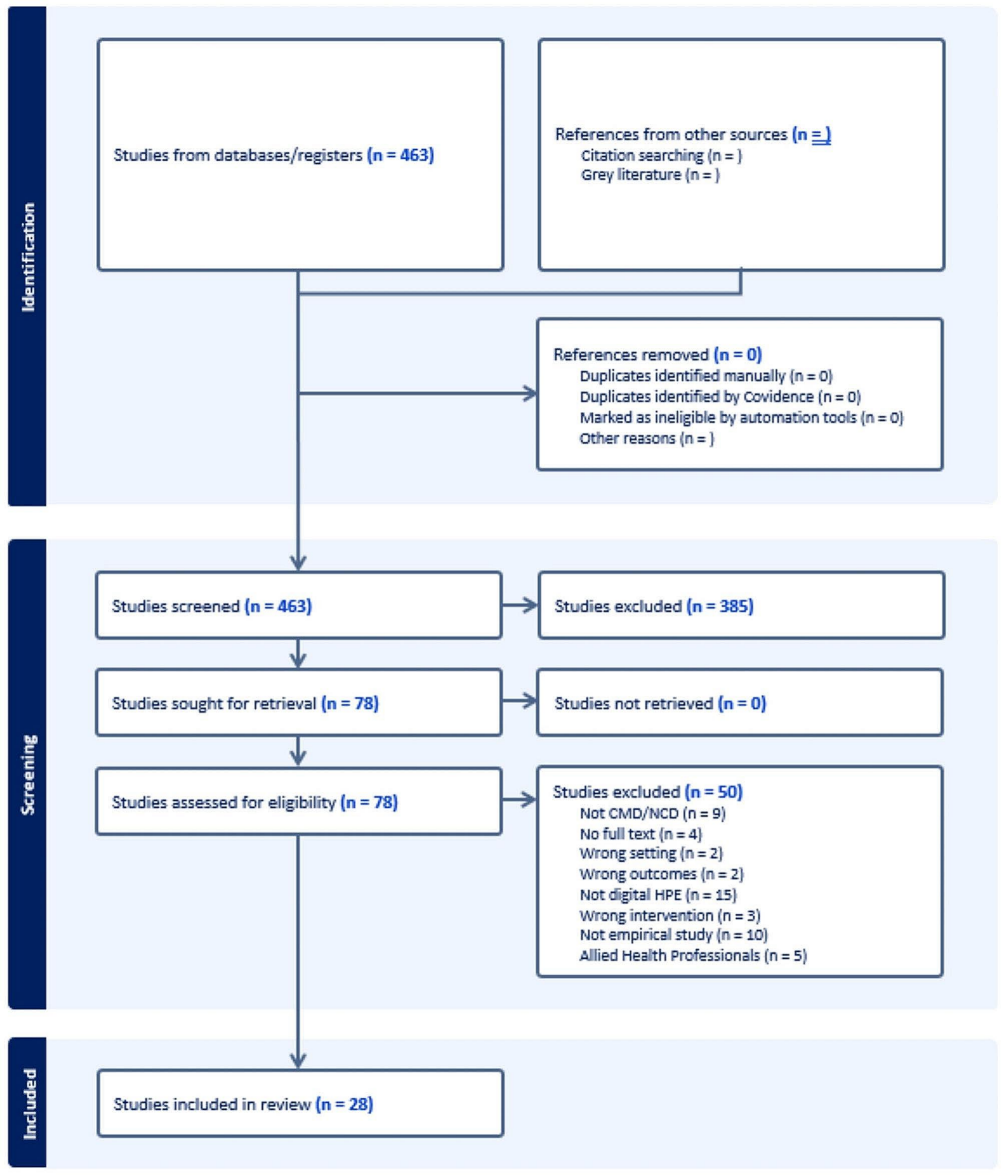
PRISMA flow chart showing the screening process

Figure 1. PRISMA flow chart showing the screening process.

In Covidence, the first step was to screen the title and abstract of these 463 documents to determine whether they were suitable for inclusion in the review. This screening process excluded studies that were.

- Not empirical (e.g., reviews and commentaries).
- About training patients to manage their own chronic disease.
- About digital health solutions (e-health, m-health, apps, etc.)
- Not related to NCD prevention, treatment, or care.

This process led to the exclusion of 385 documents, leaving a pool of 78 for full-text screening. The full-text screening followed the same exclusion criteria. This led to the exclusion of a further 50 documents, leaving a pool of 28 documents for inclusion in the review. The PRISMA flow chart in Fig. 1 illustrates this process, and Table 1 presents an overview of the 28 included studies. We note quality assessments are not typically recommended or conducted with scoping reviews [19] Moreover, as we were primarily focused on understanding what kinds of evidence exist, we did not undertake a quality assessment of the included documents.

**Table 1:** The 28 studies included in the review

Author/s (year)	Study Title	Objective
Bermejo-Caja et al. (2019) [20]	Acceptability and feasibility of a virtual community of practice to primary care professionals regarding patient empowerment: a qualitative pilot study	To pilot the acceptability and feasibility of a virtual community of practice aimed at improving the attitudes of primary care professionals to the empowerment of patients with chronic conditions
Block et al.(2018) [21]	Promoting sustainability in quality improvement: an evaluation of a web-based continuing education program in blood pressure measurement	To evaluate whether a web-based continuing education program in blood pressure measurement reinforced knowledge and skills among clinical staff and promoted sustainability of an existing quality improvement program
Conte et al. (2017) [22]	Development of A Platform For E-Training/E-Learning for Echocardiography Practitioners	To present a platform for e-training, specifically addressed to echocardiography practitioners, aiming at improving effectiveness in the clinical interpretation of echocardiography images.
De Silva et al. (2022) [23]	Utilization of an Online Education Module and Standardized Patient Simulation Experience to Improve Nursing Student Learning Outcomes with Heart Failure Patients	To examine whether nursing students ‘participation in an online comprehensive heart failure educational module enhances their knowledge and ability to apply heart failure education skills in a simulated standardized nurse-patient interaction.
Engelman et al. (2017) [24]	Evaluation of Computer-Based Training for Health Workers in Echocardiography for RHD	To measure the change in the knowledge and confidence of a group of health workers after a computer-assisted training intervention in basic echocardiography for rheumatic heart disease
Franchi et al. (2019) [25]	Simulation-Based Education for Physicians to Increase Oral Anticoagulants in Hospitalized Elderly Patients with Atrial Fibrillation	To determine whether a simulation-based education addressed to physicians was able to increase the proportion of hospitalized elderly with atrial fibrillation prescribed with oral anticoagulants compared with the usual practice
Fuchs et al. (2018) [26]	Self-learning of point-of-care cardiac ultrasound, can medical students teach themselves?	To evaluate if the training process of Point-of-care ultrasonography can be simplified by allowing medical students self-train themselves with a web-based cardiac ultrasound software
Gorchs-Molist et al. (2020) [27]	An Online Training Intervention on Prehospital Stroke Codes in Catalonia to Improve the Knowledge, Pre-Notification Compliance and Time Performance of Emergency Medical Services Professionals	To evaluate if the training process of Point-of-care ultrasonography can be simplified by allowing medical students self-train themselves with a web-based cardiac ultrasound software
Hayer et al. (2022) [28]	Using web-based training to improve accuracy of blood pressure measurement among health care professionals: A randomized trial	To evaluate the effectiveness of the e-Learning module in changing blood pressure measurement knowledge and clinical skills
Herbert et al. (2021) [29]	Developing a Smartphone App with Augmented Reality to Support Virtual Learning of Nursing Students on Heart Failure	To develop an Augmented Reality (AR) app on heart failure for remote training of nursing students and compare it against recorded video lecture using a quasi-experimental study using pre-test- post-test design with junior nursing students.
Hicks and Murano(2017) [30]	Online nutrition and T2DM continuing medical education course launched on state-level medical association	To determine whether a 1-hour online continuing medical education (CME) course focused on nutrition for type 2 diabetes would result in a gain in nutrition knowledge by practicing physicians.
Hjorth-Johansen et al. (2019) [31]	E-learning or lectures to increase knowledge about congenital heart disease in infants: A comparative interventional study	To create, implement and evaluate an e-learning course on nursing infants with congenital heart disease (CHD) and to measure its efficacy com‐pared with classroom learning
Jameison (2020) [32]	Cardiac physiology: comparison of an e-learning and classroom-based resource for first-year medical students.	To assess the efficacy and enjoyability of an e-learning resource (ELR) and a classroom-based lesson (CBL) aimed at increasing students’ understanding of cardiovascular disease by using ‘patient’-centred activities.
Kailin et al. (2021) [33]	Online Learning and Echocardiography Boot Camp: Innovative Learning Platforms Promoting Blended Learning and Competency in Paediatric Echocardiography	To evaluate the impact of online learning combined with lecture-based and hands-on teaching on the acquisition of paediatric echocardiography knowledge and confidence in paediatric cardiology and paediatric critical care fellows.
Lombardi et al. (2018) [34]	Raising Awareness of Acute Kidney Injury: A Latin American Experience	To describe the design, implementation, and results of two different online courses about acute kidney injury (AKI) targeting nephrologists and related specialists and primary care physicians. To evaluate the impact that this educational tool has had on medical knowledge.
Lukaschek et al. (2019) [35]	Applicability of Motivational Interviewing for Chronic Disease Management in Primary Care Following a Web-Based E-Learning Course: Cross-Sectional Study	To report participants’ opinion on the practicality of Motivational Interviewing for chronic disease management in primary care as learned in a web-based E-learning course, stratified by the level of education.
Okuroğlu and Alpar (2019) [36]	Effect of web-based diabetes training program on diabetes related knowledge, attitudes, and skills of health professionals: A randomized controlled trial	To determine the effect of a Web-Based Diabetes Training Program (WB–DTP) on the diabetes-related knowledge, attitudes, and skills of health professionals.
Padilha et al. (2021) [37]	Easiness, usefulness and intention to use a MOOC in nursing	To assess students and nurses’ easiness, usefulness and intention to use a Massive Open Online Course (MOOC) as an educational resource to enhance self-management intervention skills in Chronic Obstructive Pulmonary Disease patients.
Paul et al. (2017) [38]	Poor uptake of an online intervention in a cluster randomized controlled trial of online diabetes education for rural general practitioners	To describe the uptake of a continuing medical education intervention targeting diabetes management for rural general practitioners and its impact on the viability of a cluster randomised controlled trial of the effects of continuing medical education on whole-town diabetes monitoring and control.
Phuangngoenmak et al. (2019) [39]	Effectiveness of the Strengthening Diabetes Care Program: A Randomized Controlled Trial with Thai Nurse Practitioners	To examine the effects of a strengthening diabetes care program among Thai nurse practitioners working in a diabetic clinic at primary care units in a province in northern Thailand
Piya et al. (2022) [40]	The impact of nursing staff education on diabetes inpatient glucose management: a pilot cluster randomized controlled trial	To compare diabetes outcomes in medical wards where nursing staff were offered one face-to-face (F2F) session followed by access to online education (online), F2F education only, or standard care (control).
Rhodes et al. (2019) [41]	Rapid E-Learning for professional development in school-based diabetes management.	To assess the effectiveness of a rapid e-learning module for school nurse professional development in school-based diabetes management
Santiago et al.(2021) [42]	Digital educational technology for care management of diabetes mellitus people’s feet	To develop and validate a distance learning course aimed at the pillars of care management of diabetes mellitus people’s feet.
Siddiqui et al. (2018) [43]	Facebook as a Learning Tool: Perception of Stroke Unit Nurses in a Tertiary Care Hospital in Islamabad	To obtain the perception of nurses on the use of Facebook as a learning tool.
Suppan et al. (2021) [44]	Asynchronous distance learning of the National Institutes of Health Stroke Scale during the COVID-19 pandemic (e-learning vs. video): Randomized controlled trial	To determine whether an e-learning module could improve asynchronous distance knowledge acquisition of the National Institutes of Health Stroke Scale (NIHSS) in senior medical students compared to the traditional didactic video.
Tseng et al. (2021) [45]	Effectiveness of applying clinical simulation scenarios and integrating information technology in medical-surgical nursing and critical nursing courses	To determine the impact of combining clinical simulation scenario training and Information Technology Integrated Instruction (ITII) on the teaching of nursing skills.
Walker et al. (2021) [46]	Democratizing type 1 diabetes specialty care in the primary care setting to reduce health disparities: project extension for community healthcare outcomes (ECHO) T1D	To demonstrate the feasibility of an Extension for Community Healthcare Outcomes (ECHO) program focused on Type 1 Diabetes and improve primary care providers’ abilities to manage patients with Type 1 Diabetes.
Wewer Albrechsten et al. (2017) [47]	Health care professionals from developing countries report educational benefits after an online diabetes course	To investigate if participation in a 6-week open online course in the prevention and treatment of diabetes and obesity had any impact on the knowledge, skills, and career of health care professionals contrasting participants from developing countries versus developed countries.

From each of these 28 papers, we extracted data about the study’s objectives, location, target population, research design and methodology, findings, health focus, and modality of the digital educational intervention. This extraction process was undertaken by one author (SH). A few unclear cases were discussed with a further two authors (AB, LXJ). In the results section below, we present a synthesis of the extracted data, with an emphasis on the benefits and challenges identified in the various digital educational interventions.

## Results

### Description of studies

The final list of the 28 studies included in our review consisted of 22 studies from high-income countries with the majority of them from United States of America (USA). Only six studies were from LMICs, more specifically from Brazil, Pakistan, Türkiye, and Uganda, as well as two studies that spanned several LMICs.

The target groups were mostly in-service health professionals but a considerable number of studies also included pre-service students of medicine (*n* = 6) and nursing (*n* = 6). Among the targeted in-service health professionals, most were nurses (*n* = 12), followed by doctors (*n* = 8) and other health professionals (*n* = 8) including emergency technicians, primary care providers, medical assistants, etc.

**Table 2:** Study location, target population, study design, and health focus of the 28 included studies. The parenthesis after the study location signifies whether the location is in a high-income country (HIC) or in an LMIC

Author/s (year)	Study Location	Target Population	Study Design	Health Focus
Bermejo-Caja et al. [20]	Spain (HIC)	Doctors and nurses	Observational	Chronic conditions
Block et al. [21]	USA (HIC)	Medical assistants, nurses	Evaluation	Hypertension
Conte et al. [22]	Italy (HIC)	Doctors	Observational	Cardiovascular disease
De Silva et al. (2022)	USA (HIC)	Nursing students	Experimental	Cardiovascular disease
Engelman et al. [24]	Uganda (LMIC)	Health workers, nursing students, and nurses	Experimental	Cardiovascular disease
Franchi et al. [25]	Italy (HIC)	Doctors	Randomized Control Trial	Cardiovascular disease
Fuchs et al. (2018)	Israel (HIC)	Medical students	Experimental	Cardiovascular disease
Gorchs-Molist et al. [27]	Spain (HIC)	Emergency technicians, nurses, and doctors	Observational	Cerebrovascular
Hayer et al. [28]	USA (HIC)	Doctors, nurses, physician assistants, and medical assistants	Randomized Control Trial	Hypertension
Herbert et al. [29]	USA (HIC)	Nursing students	Experimental	Cardiovascular disease
Hicks and Murano [30]	USA (HIC)	Doctors	Experimental	Diabetes
Hjorth-Johansen et al. [31]	Norway (HIC)	Nursing students and nurses	Randomized Control Trial	Cardiovascular disease
Jameison [32]	UK (HIC)	Medical students	Experimental	Cardiovascular disease
Kailin et al. [33]	USA (HIC)	Medical students	Evaluation	Cardiovascular disease
Lombardi et al. [34]	Latin America (LMIC)	Doctors	Experimental	Renal
Lukaschek et al. [35]	Germany (HIC)	Doctors	Observational	Cardiovascular disease
Okuroğlu and Alpar [36]	Türkiye (LMIC)	Nurses, midwives	Randomized Control Trial	Diabetes
Padilha et al. [37]	Portugal (HIC)	Nursing students and nurses	Observational	Chronic Obstructive Pulmonary Disease
Paul et al. [38]	Australia (HIC)	Doctors	Randomized Control Trial	Diabetes
Phuangngoenmak et al. [39]	Thailand (LMIC)	Nurses	Randomized Control Trial	Diabetes
Piya et al. (2022)	Australia (HIC)	Nurses	Randomized Control Trial	Diabetes
Rhodes et al. [41]	USA (HIC)	School nurses	Observational	Diabetes
Santiago et al. [42]	Brazil (LMIC)	Nurses	Observational	Diabetes
Siddiqui et al. [43]	Pakistan (LMIC)	Nurses	Observational	Cerebrovascular
Suppan et al. (2021)	Switzerland (HIC)	Medical students	Randomized Control Trial	Cerebrovascular
Tseng et al. [45]	Taiwan (HIC)	Nursing and medical students	Experimental	Cardiovascular disease
Walker et al. [46]	USA (HIC)	Doctors	Observational	Diabetes
Wewer Albrechsten et al. (2017)	Various “developing countries” (LMIC)	Doctors, nurses, midwives, and medical students	Observational	Diabetes

The majority of the studies in the overall pool used either experimental or observational study designs and gathered data using online questionnaires, interviews, and/or analysis of individual or online interactions between learners. The details about target groups and study designs are shown in Table 2. We use the term *experimental* for studies that have no specific information on the randomization of the participants or where randomization has not been done. These studies typically included two groups of the study population, where one group served as an experimental one provided with the intervention and the other with no or some traditional type of intervention. Other than the observational and experimental studies, randomized control trials (RCTs) and evaluation studies were part of our review.

The studies in our review comprised mainly of educational interventions related to diabetes, stroke, hypertension and cardiac disorders.

### Assessment of digital educational intervention

Based on the digital education modality that was described, we grouped the studies into three categories: blended learning, online learning with instructor, and online learning as self-study. In the sub-sections below we present the interventions, study findings, effectiveness and identified challenges of each modality.

### Blended learning

Our review includes seven studies providing blended learning to health professionals and students. For this purpose, we identify blended learning as any intervention that combines online learning with some form of onsite training or teaching. All the studies report the advantages of blended learning over traditional learning and the increase in overall knowledge.

Blended learning was incorporated in various formats in the studies. Some of the studies include the online learning proponent prior to the onsite training [33, 40]. In these, the online learning was provided in modules that could be taken at the participants’ own pace before the onsite programme which was characterised by hands-on workshops and lectures. Other studies began with on-site training followed by an online learning proponent [23, 36, 39]. In these studies, the online proponent consisted of further self-study of the content learned in the prior onsite training. The remaining two studies did not have a set order but rather had the online proponent as a learning resource that the participants could draw upon among other resources such as tele-education sessions, a local support coach [46] or interactive classroom lectures with group discussions and role play [43].

The studies consisted of both RCTs and observations. The RCT studies mostly highlighted the strengthening capacity of nursing professionals. For instance, in one RCT study in Thailand, the findings showed the effectiveness of blended learning in strengthening competency in diabetes care among nurses, wherein the levels of perceived self-efficacy, outcome expectancy, knowledge and skills in diabetes management care were statistically and significantly higher at Weeks 4 and 8 compared to the control group [39]. In another RCT conducted in Australia, the addition of access to online learning, as well as face-to-face education, significantly increased the uptake of diabetes education among hospital non-specialist nursing staff [40]. A study based in Pakistan gathered information about perceptions about social media as a tool for online training and reported that Facebook, with tutor support, enabled participants to study the material when their schedule permitted. The online teaching component and facilitation were ideal for their full-time working nurses, as reflected by their improved post-course test results [43]. The detailed findings for studies examining blended learning are provided in Table 3.

Generally, among health professionals, the perception of blended learning was positive. Blended learning was perceived to be beneficial and impactful in increasing knowledge. This type of learning makes the learning interactive. However, certain challenges were identified that hampered online learning, e.g., limited internet connection and computer skills for the participants enrolled in the learning [43]. As many of the participants are health professionals active in the workforce, the long duration of the working hours makes it difficult to spare time for online learning [36, 40].

**Table 3:** Findings of studies involving blended learning

Author/s (Year)	Online Learning Tool	Findings
De Silva et al. (2022)	Web based LMS (HFEM)	The findings from this research indicate that large gains can be made in both student knowledge attainment and knowledge application if educators move beyond normal classroom/lecture teaching techniques. The total mean of 17.99 (SD = 1.37) for the experimental group on the heart failure posttest was significantly higher, F (1,142) = 408.74, *p* < 0.01, than the control group total mean score of 12.69 (SD = 1.77).
Kailin et al. (2021)	Web based LMS (pedecho)	Benefits to online learning modules was demonstrated for the short and medium-term retention of echocardiographic learning content. The online learning group demonstrated improvement in exam scores following online learning (PRE 49.1 ± 15.3 vs. POST 67.8 ± 17%; *p* ≤ 0.01). Medical learners from around the world who had not been previously exposed to echocardiographic learning content exhibited a clear benefit from this online platform and learning materials
Okuroğlu and Alpar (2019)	Web based LMS	It was determined that the Web based distance training program was effective at increasing the diabetes-related knowledge and skills of the healthcare professionals. The scores of the achievement test post-test (*P* < 0.001) and follow-up test (*P* < 0.001) were significantly higher in the intervention group, compared to the control group that receiving face-to-face education from a diabetes nurse. However, the program was not adequate for enhancing the diabetes-related attitudes of health professionals, as the diabetes-related attitudes were similarly positive (> 3) for both groups with no significant difference.
Phuangngoenmak et al. (2019)	Web based LMS	The findings showed the effectiveness of the program in strengthening competency in diabetes care among nurses. In the experiment group, this program significantly increased perceived self-efficacy, outcome expectancy, knowledge and skills in Diabetes Mellitus care at Weeks 4 and 8, compared to the baseline (all *p* < 0.003). In addition, when compared to the control group, the levels of perceived self-efficacy, outcome expectancy, knowledge and skills in Diabetes Mellitus care were statistically and significantly higher at Weeks 4 and 8 (all *p* < 0.02).
Piya et al. (2022)	Web based LMS	The online ward, but not the Face-to-Face (F2F) or control wards (control group) showed increased number of good diabetes days (GDD). In the online ward, GDD improved from 4.7(2.7–7.0) to 6.0(2.3–7.0) days; *p* = 0.038. However, there was no difference in length of stay (LOS) change between online [Median (IQR) 5 (2–8) to 4 (2–7) days], F2F [7 (4–14) to 5 (3–13) days] or control wards [5 (3–9) to 5 (3–7) days].
Siddiqui et al. (2018)	Facebook	Facebook enabled participants to study the material when their schedule permitted it. The online teaching and facilitation were ideal for our full-time working nurses as reflected by their improved post-course test results. The post-course test showed that nine of 10 candidates passed with scores > 70% compared to only two candidates getting scores > 50% in the pre-course test.
Walker et al. (2021)	Web based LMS (ECHO T1D)	Following the pilot study, there was statistically significant improvement in primary care providers’ diabetes knowledge (*p* ≤ 0.01) as well as in diabetes confidence (*p* ≤ 0.01). The results of this pilot study demonstrate that the blended learning addresses the gaps by equipping more practitioners with the knowledge and resources to support patients with Type 1 diabetes who may not otherwise receive adequate or routine specialty care.

### Online learning with instructor

There were six studies in our review, wherein online learning with instructors was explored. Such online learning includes following a simultaneous schedule allowing for contact between learners and teachers/trainers during the course. Two of the six studies had no control group. All the studies assessed the effects of their online teaching through survey-based questionnaires. A majority of the studies reported that these types of courses are cost-effective and can help bypass the geographical barrier. The findings of these studies are given in Table 4.

Regarding instructor involvement, five of the studies used learning platforms such as Moodle or Zuvia for the instructor to organise courses, materials and activities [22, 27, 38, 42, 45]. Four of these also had an online forum or messaging app for peer discussions about the content, two of these also included interactions with faculty and tutor support [27, 38, 42, 45]. For instance, a study by Paul et al. [38] had an online request form for specialist advice regarding diabetes. The last study by Hicks and Murano [30] had an instructor-led webinar followed by self-study.

The studies showed a positive effect on practice. A Spanish study on cerebrovascular medical emergency management from reported that interprofessional online stroke training in the Catalonian Emergency Medical Service (EMS) was effective in increasing the study participants’ knowledge of cerebrovascular medical emergencies. The results encouraged the Catalonian EMS to maintain this training intervention in their continuous education program [27].

**Table 4:** Findings of studies involving online learning with instructor

Author/s (Year)	Online Learning Tool	Findings
Conte et al. (2017)	Web based LMS & Smartphone App	The authors present the E-Learning part as its useful to introduce the E-Training and is considered, as a real innovation of the overall tool. The E-Training represents a challenging way to update knowledge also in expert users, to improve their skills and to compare their opinion with the whole peer-community and with the coordination team over frequently updated tricky clinical cases
Gorchs-Molist et al. (2020)	Web based LMS (Moodle)	An interprofessional online training intervention on strokes in the Catalonian Emergency Medical Services was effective in increasing the participant’s knowledge on cerebrovascular medical emergencies. There was a significant increase in 80% of the questions regarding recognition of signs and symptoms of a stroke (*p* < 0.05), and a significant increase in all questions regarding stroke code, prehospital management and stroke assessment, with most scores above 85% (*p* < 0.001). Both strengths and areas for improvement were detected for future training opportunities. These results encouraged the Catalonian Emergency Medical Services to maintain this training intervention in their continuous education program, which, starting back in 2015, is delivered twice a year.
Hicks and Murano (2017)	Web based LMS	This research study demonstrated that online continuing medical education courses launched on state-level medical association platforms improved subject matter knowledge significantly between the pre- and post-scores (*P* < 0.0001). Ultimately, doctors who couple knowledge basics with practical application tools may more successfully integrate these into their practices.
Paul et al. (2017)	Web based LMS	Given that the number of doctors completing the online learning module was too few to expect an intervention effect, the intervention trial was discontinued, that is, no follow-up data were collected.
Santiago et al. (2021)	Web based LMS (Moodle)	It was possible to develop a digital educational technology for care management of Diabetes Mellitus people’s feet.
Tseng et al. (2021)	Web based LMS	For student performance, there was no significant difference between the experimental and control groups before the course, demonstrating their similarities, but student performance for the knowledge component in the experimental group (innovative instruction) was better than the control group (conventional instruction) after the courses. The Objective structure clinical examinations (OSCE) total score for the experimental group was M = 230.18, SD = 26.89 vs. M = 194.97, SD = 21.66 for the control group (*p* < 0.001). This, demonstrating the effectiveness of innovative instruction to boost knowledge-based learning within a short amount of time.

### Online learning as self-study

Of the included papers, 15 were about online learning as self-study. In such an intervention, the learner undertakes an online course/training as flexible self-study. This means the course can be done at any time and does not require any set schedule or contact with teaching staff. Table 5 presents an overview of the study findings.

Largely the studies using online learning as self-study reported improvements in learning following the training. For instance, A study across Latin American countries studied the effects of online training on medical knowledge regarding acute kidney injury (AKI) on nephrologists and primary care physicians. The study reported gains in knowledge equivalent to 36%. It is important to note that the study concluded that the interactive, asynchronous, online courses were valuable and successful tools for continuing medical education in Latin America, reducing heterogeneity in access to training across countries. The application of distance education techniques has proved to be effective, not only in terms of primary learning objectives but also as a potential tool for the development of a sustainable structure for communication, exchange, and integration of physicians and allied professionals involved in the care of patients with AKI [34]. However, one study explored the use of online simulations [25]. This randomized control trial reported no significant change in the experimental group following an online educational course regarding oral anticoagulants in case of atrial fibrillation. Also, the reading material in certain modules being too dense and lengthy poses a challenge for the participants in one study to complete the learning [45]. Another study by Lombardi et al. [34]., also questioned whether the knowledge effect is retained on a long-term basis.

Some of the studies emphasise the possibilities that online learning provides. One study indicated that a 6-week internet-based course in diabetes and obesity treatment may serve as an important resource in postgraduate education for medical doctors as well as other health professionals. From a wider perspective, education based on Massive Open Online Courses (MOOC) may assist the professional community by providing the latest evidence-based guidelines in an easily accessible and globally available way [47]. An evaluation study in the United States reported that online learning modules can be developed and maintained with minimal costs and basic technological requirements and present a unique opportunity to provide essential information in a short timeframe. In addition, these modules can be specifically tailored to address identified knowledge gaps among various groups and can be easily disseminated and can be an effective method for educating nurses in a time- and cost-sensitive manner [41].

The major challenges faced by health professionals or students when participating in online learning by self-study include time constraints and out-of-date or inappropriate hardware and software [20, 34]. Some barriers that online learning can help organisations overcome include logistical difficulties and expenses associated with maintaining an adequate pool of educators, coordinating training sessions, and standardizing training across sites [21].

**Table 5:** Findings of studies involving self-study

Author (Year)	Online Learning Tool	Findings
Bermejo-Caja et al. (2019)	Web based LMS (e MPODERA)	The results from this pilot study show qualitatively that primary care professionals considered the e-learning application useful for learning how to empower patients. However, attention needs to be paid to technological issues, and the time demands on professionals.
Block et al. (2018)	Web based LMS (Learnshare)	Direct observation data revealed that completing the online training program was associated with improvement in certain steps in the blood pressure protocol, including explaining the protocol to patients, providing a rest period, use of average mode, and recording the average reading in the EMR. Prior to the module, the participants answered 80.6% of questions correctly and after the module, they answered 93.4% correctly (*p* < 0.01), and improvements were significant for staff from all job types.
Engelman et al. (2017)	Web based LMS	In this evaluation of computer-assisted training materials for Rheumatic Heart Disease screening, strong evidence was reported for an increase in knowledge across all required learning areas. They reported an increase in the mean total score on knowledge tests from 44.8–85.4% (mean difference: 40.6%, 95% confidence interval: 35.4 to 45.8%). Additionally, increased confidence in core competencies for a group of health workers without previous training in ultrasonography was also reported with a significant increase in confidence scores clinical science, echocardiography and overall (*p* < 0.001 for all three scales). Use of the training modules may reduce face-to-face teaching times and therefore the human resource requirements for busy clinicians and faculty.
Franchi et al. (2019)	Web based LMS (Dr Sim)	In this trial conducted in internal medicine and geriatric wards, an educational course based on a computer-based simulation did not succeed in obtaining an increase in the proportion of patients with Atrial Fibrillation prescribed with any Oral Anti Coagulants at hospital discharge with respect to the usual practice (odds ratio, 1.46; 95% confidence interval, 0.81–2.64). However, in the intervention arm, there was a greater increase, compared to the control arm, in the proportion of patients prescribed with Oral Anti Coagulants (15.1%; 95% Confidence interval, 0-31.5%) and with direct oral anticoagulants (20%; 95% confidence interval, 0-39.8%).
Fuchs et al. (2018)	Web based LMS (eMedical Academy)	This study shows that medical students were able to independently learn how to acquire cardiac ultrasound views by using an e-learning platform in combination with self-practice. Students who trained on their own, with no bedside teaching, combining an e-learning module and self-cardiac ultrasound practice, were overall as good as students who received an already validated, bedside, frontal cardiac ultrasound course with no significant difference between the two groups (*p* = 0.508).
Hayer et al. (2022)	Web based LMS	Across all four sites, participants in the intervention group demonstrated significant improvement in both their knowledge and skills after completing the 30-minute e-Learning module. Following the e-Learning module, the intervention group performed on average 3.4 more objective structured clinical examination items and 1.7 more knowledge questions correctly vs. 1.4 and 0.5 in the control group following a test-rest approach (*P* < 0.01). The intervention group also had a 17.1% increase in the obtainment of accurate systolic and diastolic blood pressure measurement compared to a 6.1% decline in the control group (*p* < 0.01). The results confirm the need for BP measurement retraining and the ability to improve provider’s knowledge and skills with a brief 30-minute e-Learning module.
Herbert et al. (2021)	Smartphone Augmented Reality (AR) App	When comparing the overall HFA pre-test and post test results, no key learning differences were identified for either the experimental or recorded video lecture control groups in both the assessment completion time (t [3] = 1.626, *p* = 0.114) and overall % test accuracy (t [30] = 1.846, *p* = 0.075). Overall performance across groups was not as high as the authors expected.
Hjorth-Johansen et al. (2019)	Web based LMS	While the scores between the control group (face-to-face learning) and the intervention group (e-learning) did not differ significantly, both groups improved their scores significantly. The score of the face-to‐face learning group (control) increased significantly from 22.9–36.5 (*p* < 0.001), while that of the e‐learning group increased from 27.8–38.0 (*p* < 0.001). The fact that e-learning was the less time-consuming learning method and that traditional learning was the preferred learning method may imply that blended learning, mixing traditional learning and e-learning may be the most effective learning method. This may address both the need for time effectiveness and the need for interaction with a teacher or an expert.
Jameison (2020)	Web based LMS (SoftChalk)	The study met its initial aims and significantly improved participant’s confidence in their understanding of cardiovascular disease, with a greater mean increase in confidence across all topics from the e-learning resources than the classroom lessons (Scores of 2.46 vs. 1.80). The study suggests e-learning and student led classroom-based learning are effective methods of teaching, with students commenting that both were engaging and enjoyable.
Lombardi et al. (2018)	Web based LMS	Before and after the online course for nephrologists, the mean number of right answers was 5.87 and 8.01, respectively (*P* < 0.05). The pretest and posttest scores for the primary care physicians were not reported. The interactive, asynchronous, online courses are a valuable and successful tool for continuing medical education in Latin America, reducing heterogeneity in access to training across countries. Reliable information is lacking regarding the impact of these courses on long-term knowledge retention and the ultimate benefit on quality of health.
Lukaschek et al. (2019)	Web based LMS (EQuiP)	The knowledge and skills obtained by the Web-based motivational interviewing course were assessed by the participants as being beneficial and appropriate for use in primary care practice. Regarding the perceived applicability of skills and knowledge from the course, the groups rated the following: medical students: 94% [79/84] *good*; Physicians in specialist training: 88.6% [109/123] *excellent*; and General practitioners: 51.3% [182/355] *excellent*.
Padilha et al. (2021)	Massive Open Online Courses (MOOC)	This study shows that independently of age, nurses consider MOOC useful for education and lifelong learning, expressing usefulness and intention to use this educational strategy in the future. Two dimensions presented average scores near the maximum scale value, the dimension 1 (F1) - Easiness and global quality of the course (M = 4.70, SD = 0.314) and dimension 2 (F2) - Usefulness and intention to use in the future this type of course (M = 4.73, SD = 0.346). Regarding the easiness of use and global quality of the MOOC, specialised nurses, who are older and with expertise in this field, scored higher than nurses and students. These data show that the MOOC is not only directed to younger generations but most importantly are highly beneficial to those who need access to education and lifelong learning to keep up to date.
Rhodes et al. (2019)	Web based LMS	The results of the present study suggest that these modules can be an effective method for educating school nurses in a time- and cost-sensitive manner. The independent sample t-test revealed a statistically significant (*p* < 0.001) difference between pre-test (M = 12.79, SD = 2.05) and post-test (M = 17.15, SD = 2.00) scores (t (1125) = − 35.19, *p* = 0.00).
Suppan et al. (2021)	Web based LMS	Asynchronous distance learning using a highly interactive e-learning module yielded better results than following the traditional didactic video on the web (38 correct answers, 95% CI 37–39, vs. 35 correct answers, 95% CI 34–36, *P* < 0.001).
Wewer Albrechsten et al. (2017)	MOOC (Coursera)	Over 80% of the health care participants report educational benefits, improved knowledge about the prevention and treatment therapies of diabetes and furthermore improved professional life and practice. Participants from developing countries gained more impact on their clinical practice (94%) compared to health care professionals from developed regions (88%) (Mean of differences = 6%, *P* = 0.03. The results indicate that a 6-week internet-based course in diabetes and obesity treatment may serve as an important resource in postgraduate education for medical doctors as well other health care professionals.

## Discussion

This section discusses the strengths, weaknesses, and advantages of digital education related to NCDs in the reviewed literature in the context of India.

### Value of online and blended NCD education

The limited literature available on the topic paints a positive picture regarding the increase in learning/knowledge of health professionals on NCDs due to online learning. A majority of the studies reported an increase in knowledge after the interventions. A study from Latin America provides an example of how online courses can be a valuable and successful tool for continuing medical education and reducing heterogeneity in access to training across countries. The diverse findings suggest that modality alone is not the sole issue; for example, a recent study comparing traditional vs. online learning [44] suggests interactivity may matter.

The studies reported a number of challenges related to the online format in general. One highlighted that training of healthcare providers can be more difficult in time constrained and low-resource settings due to limited accessible equipment, inadequate environment and competing interests [28]. Another found that augmented reality smartphone apps may not provide the extensive information needed for complex content [29]. The senior doctors were not as pleased as their less-experienced colleagues with the web-based format of the learning [35]. Online training options, while notionally attractive and accessible, are not likely to have high levels of uptake as they require more commitment, activity, and dedication [38]. Although there are challenges with online learning, the included studies also emphasized the opportunities it provides, e.g. making knowledge more accessible to a wider population and making it more flexible for health professionals with heavy workloads to learn at their own pace [36, 39, 47].

Although we categorize and present the interventions in the three modalities, it is important to note that many of the challenges and opportunities we found are shared by all modalities. Because of this, it is not possible to highlight a single modality that is best in all situations – rather, they each have different affordances in relation to important considerations such as learner flexibility or programme scalability. Online learning as self-study offers almost complete learner flexibility and programme scalability – but it lacks important elements of individualized feedback, collaborative learning, and the motivation that learners and teachers can experience when they are together in the same room at the same time. Blended learning tries to balance the advantages of being together with the flexibility of learning online. This blend can take many forms, and rather than a single pedagogical approach, it should probably be considered a spectrum of approaches inhabiting the space between campus learning and online self-study.

### Relevance to Indian context

The review showed that most of the literature is from high-income countries like the United States, United Kingdom, Australia, and Spain. Only very few studies describe educational interventions set in LMICs, and none of them were from India. It is, however, important to point out that the category LMIC is very broad, including both countries in sub-Saharan Africa, as well as countries like Türkiye and Thailand. This entire spectrum is also present within India. Despite the great diversity within India, the high-income setting of most of the described interventions limits their direct applicability in many of the most underserved Indian contexts, where the health professions, education systems, and health care systems in general already have significantly fewer resources. We hope, however, that the experiences from other countries can serve as inspiration for educational interventions and research which is tailored to the needs, challenges, and opportunities that are relevant to India.

In an Indian context, the main advantage of online learning is the flexibility to reach people in rural areas, especially for in-service training of health professionals who are no longer residing close to a medical or nursing college. This flexibility is even more pronounced with online self-study training. The advantages of online learning are beginning to be recognised in India. During the last decade, the digital education platform has seen a perceptible growth in India. Several public and private organizations and entities have started providing digital training for capacity building of healthcare professionals especially in terms of NCDs. Different types of courses are offered in the form of online or blended learning. However, it is important to note, that the use of digital education and training in rural areas comes with its own set of challenges in relation to lacking connectivity and insufficient technical infrastructure. Furthermore, the significant linguistic and cultural diversity of India, also influences how well digital education interventions can scale. Nonetheless, with the NEP 2020 focusing on digital and equitable education among health care professionals and the post-pandemic time period, the courses offered digitally have increased severalfold. Introducing such courses in The National Programme for Prevention & Control of Non-Communicable Diseases (NP-NCD) could help India address shortages and skewed distributions of its health workforce. Also, with the introduction of MOOCs and EdTech investments in the last decade, many leading universities and schools of public health are hosting NCD courses, which are available for learners in the Indian subcontinent and worldwide. These are primarily aimed at medical doctors, with just very few targeting nurses. Many of the courses that are open to nursing are open to almost all sections of health care workers.

Examples of digital training in India mainly focus on diabetes education and are provided by the government through public institutions as well as private organizations. Some examples of online training on diabetes through government institutions include through National Institute of Public Health Training and Research (NIPHTR) and Christian Medical College (CMC) Vellore [48, 49]. In addition to these, various organizations have partnered to provide quality training courses on diabetes. One such example is an online certification course in diabetes by British Medical Journal & Fortis C-DOC, endorsed by The Royal College of Physicians (RCP), London [50]. Another example is an online training on diabetes targeted at primary care physicians offered by Public Health Foundation of India (PHFI). PHFI has developed the capacity of more than 15,000 primary care physicians with its various diabetes-related capacity-building programs since 2010 in collaboration with academic partners like Dr. Mohan’s Diabetes Education Academy (DMDEA) [51]. There are numerous examples of online courses on diabetes education that have been started in recent times [52, 53]. However, these trainings through online learning have rarely been evaluated and there is a lack of literature examining the effectiveness of such programs.

However, India faces some challenges to online learning as well. The adherence to course curriculum and retention rates will vary according to different health professionals of different geographical regions. Technological issues like internet connectivity, limited computer skills, and out-of-date software or hardware can have direct effects on the participation of health professionals. Also, there might be reluctance in the case of senior professionals to learn from their junior colleagues in instructor-based online learning [35].

### Strengths and limitations

This review is a diverse contribution from a team of Indian and non-Indian authors.

Our review includes a wide range of study designs and methodologies.

The review synthesizes evidence on an emerging topic in Lower Income Countries and provides evidence for further research.

We did not systematically employ dual independent screening and data extraction.

We did not conduct a formal assessment of the quality of the included literature. However, this is typical of scoping reviews [19], and also, the value of the insights we gained from the included studies was not necessarily bound to the quality of their findings.

To focus on current forms of digital teaching and learning we chose to limit our search to research published since 2017. Including older publications, or those in the grey literature, may have yielded further evidence that could have had relevance to our objectives.

## Conclusion

Digital education related to NCDs has proven to be beneficial for both in- and pre-service health professionals. Digital education may also offer an effective way to bypass geographical barriers that can be utilized for capacity building of the existing health workforce especially in relation to NCDs. Despite these positive attributes, and an increased openness to learning and collaborating online, digital education faces many challenges for its successful implementation in the Indian context. Owing to the multi-lingual and diverse health professional ecosystem in India, there is a need for strong evidence and guidelines based on prior research in the Indian context. Rigorous research in the form of evaluation, quasi-experimental studies or RCTs needs to be done in order to address the challenges and uncover potentials for online learning in India.

Declarations.

### Electronic supplementary material

Below is the link to the electronic supplementary material.

Supplementary Material 1

## Data Availability

All data generated or analysed during this study are included in this published article [and its supplementary information files].
