# Supplementary material for: Non-communicable diseases, digital education and considerations for the Indian context – a scoping review
Source: BMC Public Health. 2024 May 10;24:1280. doi: 10.1186/s12889-024-18765-7 (PMC11083803; doi:10.1186/s12889-024-18765-7)
Supplement: Supplementary file 1 — Supplementary Material 1 [file 12889_2024_18765_MOESM1_ESM.docx]

# Appendix 1– Search terms

**Digital education terms**

"digital education*" OR "digital learn*" OR "digital instruction*" OR "digital self-learn*" OR "digital self-learn*" OR "digital teaching" OR "digital course*" OR "digital training" OR "digital tool*" OR "digital library" OR "online learn*" OR "online instruction*" OR "online course*" OR "online training" OR "online education*" OR "online teaching" OR "e-instruction*" OR "e instruction*" OR "e-learn*" OR "e-learn*" OR "e-teaching" OR "e teaching" OR "e-resources" OR "e resources" OR "blended learn*"OR "blended instruction*" OR "blended course*" OR "blended training" OR "blended education*" OR "blended teaching" OR "distributed learn*" OR "distributed instruction*" OR "distributed course*" OR "distributed training" OR "distributed education*" OR "distributed teaching" OR "mediated learn*" OR "mediated instruction*" OR "mediated course*" OR "mediated training" OR "mediated education*" OR "mediated teaching" OR "distance education*" OR "distance instruction* OR "distance learn*" OR "distance course*" OR "distance teaching" OR "distance training" OR "virtual education*" OR "virtual classroom" OR "virtual education*" OR "virtual learn*" OR "virtual instruction*" OR "virtual course*" OR "virtual training" OR "virtual teaching" OR "remote learn*" OR "remote instruction*" OR "remote course*" OR "remote training" OR "remote education*" OR "remote teaching" OR "multimedia learn*" OR "multimedia course*" OR "multimedia instruction*" OR "multimedia training" OR "multimedia education*" OR "multimedia teaching" OR "reusable learning object*" OR "web-based learn*" OR "web-based instruction*" OR "web-based teaching" OR "web-based course*" OR "web-based training" OR "web-based education*" OR "web based learn*" OR "web based instruction*" OR "web based teaching" OR "web based course*" OR "web based training" OR "web based education*" OR "internet-based learn*" OR "internet-based instruction*" OR "internet-based teaching" OR "internet-based course*" OR "internet-based training" OR "internet-based education" OR "internet based learn*" OR "internet based instruction*" OR "internet based teaching" OR "internet based course*" OR "internet based training" OR "internet based education*" OR "digitally mediated learn*" OR "digitally mediated instruction*" OR "digitally mediated course*" OR "digitally mediated training" OR "digitally mediated education*" OR "digitally mediated teaching" OR "simulation-based learn*" OR "simulation-based instruction*" OR "simulation-based education*" OR "simulation-based teaching" OR "simulation-based training" OR "simulation-based course*" OR "simulation based learn*" OR "simulation based instruction*" OR "simulation based education*" OR "simulation based teaching" OR "simulation based training" OR "simulation based course*" OR "computer-assisted learn*" OR "computer-assisted instruction*" OR "computer-assisted course*" OR "computer-assisted training" OR "computer-assisted education*" OR "computer-assisted teaching OR "computer assisted learn*" OR "computer assisted instruction*" OR "computer assisted course*" OR "computer assisted training" OR "computer assisted education*" OR "computer assisted teaching" OR "computer-based learn*" OR "computer-based instruction*"OR "computer-based course*" OR "computer-based training" OR "computer-based education*" OR "computer-based teaching OR "computer based learn*" OR "computer based instruction*" OR "computer based course*" OR "computer based training" OR "computer based education*" OR "computer based teaching" OR "computer-aided instruction" OR "computer-aided learn*" OR "computer-aided instruction*"OR "computer-aided course*" OR "computer-aided training" OR "computer-aided education*" OR "computer-aided teaching OR "computer aided learn*" OR "computer aided instruction*" OR "computer aided course*" OR "computer aided training" OR "computer aided education*" OR "computer aided teaching" OR "mobile learn*" OR "mobile instruction*" OR "mobile course*" OR "mobile training" OR "mobile education*" OR "mobile teaching" OR "video-based learn*" OR "video-based instruction*" OR "video-based course*" OR "video-based training" OR "video-based education*" OR "video-based teaching" OR "video based learn*" OR "video based instruction*" OR "video based course*" OR "video based training" OR "video based education*" OR "video based teaching" OR "video-teleconference" OR "tele-learn*" OR "tele-instruction*" OR "tele-course*" OR "tele-training" OR "tele-education*" OR "tele-teaching" OR "tele learn*" OR "tele instruction*" OR "tele course*" OR "tele training" OR "tele education*" OR "tele teaching" OR "audio response system" OR "learning management system" OR "course management system" OR "Massive Open Online Course*"

**Health Profession terms**

medicine OR "MBBS" OR "MD" OR medical OR "med-ed" OR nursing OR nurse* OR dentistry OR dentist* OR "health professional*" OR "health professions education" OR "human resources for health" OR "healthcare workforce" OR "health worker*" OR "health workforce" OR "health education" OR "AYUSH" OR Ayurveda OR Yoga OR "Yoga and Naturopathy" OR Unani OR Siddha OR Homeopathy OR ("Indian system" AND "Medicine") OR (Indigenous AND Medicine) OR "Indian Medicine and Homeopathy" OR "ISM & H" OR "ANM" OR "Auxiliary Nurses and Midwives" OR midwives OR midwifery OR allopathy

**CMD (NCD) terms**

"cardiometabolic disease*" OR "cardiometabolic syndrome" OR "metabolic syndrome" OR "non-communicable disease*" OR "chronic disease*" OR "chronic condition*" OR "Syndrome X" OR "beer belly syndrome" OR "diabetes" OR diabetic "insulin resistance" OR "elevated fasting blood sugar" OR "impaired glucose tolerance" OR stroke* OR "cerebrovascular accident*" OR "cardiovascular disease*" OR "cardiovascular event*" OR "coronary heart disease" OR "coronary artery disease" OR "cerebrovascular disease" OR "peripheral arterial disease" OR "rheumatic heart disease" OR "deep vein thrombosis and pulmonary embolism" OR "congenital heart disease" OR "heart attack*" OR "heart failure" OR angina OR "high blood pressure" OR "abdominal adiposity" OR "acute myocardial infarction" OR "thromboembolic disease*" OR "non-alcoholic fatty liver disease*" OR "chronic renal failure" OR hyperlipidemia OR dyslipidemia OR nephropathy OR hypertension OR "hypertensive heart disease"
